# Development of a multi-electrode array for spinal cord epidural stimulation to facilitate stepping and standing after a complete spinal cord injury in adult rats

**DOI:** 10.1186/1743-0003-10-2

**Published:** 2013-01-21

**Authors:** Parag Gad, Jaehoon Choe, Mandheerej Singh Nandra, Hui Zhong, Roland R Roy, Yu-Chong Tai, V Reggie Edgerton

**Affiliations:** 1 Biomedical Engineering IDP, University of California, 90095, Los Angeles, CA, USA; 2 Neuroscience IDP, University of California, 90095, Los Angeles, CA, USA; 3 Department of Integrative Biology and Physiology, University of California, Los Angeles, Terasaki Life Sciences Building, 610 Charles E. Young Drive East, 90095-7239, Los Angeles, CA, USA; 4 Department of Neurobiology, University of California, 90095, Los Angeles, CA, USA; 5 Department of Neurosurgery, University of California, 90095, Los Angeles, CA, USA; 6 Brain Research Institute, University of California, 90095, Los Angeles, CA, USA; 7 Department of Electrical Engineering, California Institute of Technology, 91125, Pasadena, CA, USA; 8 Department of Mechanical Engineering, California Institute of Technology, 91125, Pasadena, CA, USA; 9 Department of Bioengineering, California Institute of Technology, 91125, Pasadena, CA, USA

**Keywords:** Spinal cord electrode array, Spinal cord injury, Epidural stimulation, Motor recovery

## Abstract

**Background:**

Stimulation of the spinal cord has been shown to have great potential for improving function after motor deficits caused by injury or pathological conditions. Using a wide range of animal models, many studies have shown that stimulation applied to the neural networks intrinsic to the spinal cord can result in a dramatic improvement of motor ability, even allowing an animal to step and stand after a complete spinal cord transection. Clinical use of this technology, however, has been slow to develop due to the invasive nature of the implantation procedures, the lack of versatility in conventional stimulation technology, and the difficulty of ascertaining specific sites of stimulation that would provide optimal amelioration of the motor deficits. Moreover, the development of tools available to control precise stimulation chronically via biocompatible electrodes has been limited. In this paper, we outline the development of this technology and its use in the spinal rat model, demonstrating the ability to identify and stimulate specific sites of the spinal cord to produce discrete motor behaviors in spinal rats using this array.

**Methods:**

We have designed a chronically implantable, rapidly switchable, high-density platinum based multi-electrode array that can be used to stimulate at 1–100 Hz and 1–10 V in both monopolar and bipolar configurations to examine the electrophysiological and behavioral effects of spinal cord epidural stimulation in complete spinal cord transected rats.

**Results:**

In this paper, we have demonstrated the effectiveness of using high-resolution stimulation parameters in the context of improving motor recovery after a spinal cord injury. We observed that rats whose hindlimbs were paralyzed can stand and step when specific sets of electrodes of the array are stimulated tonically (40 Hz). Distinct patterns of stepping and standing were produced by stimulation of different combinations of electrodes on the array located at specific spinal cord levels and by specific stimulation parameters, i.e., stimulation frequency and intensity, and cathode/anode orientation. The array also was used to assess functional connectivity between the cord dorsum to interneuronal circuits and specific motor pools via evoked potentials induced at 1 Hz stimulation in the absence of any anesthesia.

**Conclusions:**

Therefore the high density electrode array allows high spatial resolution and the ability to selectively activate different neural pathways within the lumbosacral region of the spinal cord to facilitate standing and stepping in adult spinal rats and provides the capability to evoke motor potentials and thus a means for assessing connectivity between sensory circuits and specific motor pools and muscles.

## Background

It is well established that the spinal cord contains intricate computing units capable of performing rapid ongoing motor processing of complex proprioceptive and cutaneous input during coordinated motor behaviors such as standing and stepping [1]. Neural networks in the lumbosacral spinal cord (i.e., central pattern generators (CPG)) can function autonomously (without any brain control) to produce the characteristic alternating motor patterns of gait and to compensate for errors and obstacles [2,3] using only sensory information from the limbs [4-7]. More recently it has become recognized that these networks have the ability to process complex sensory ensembles that can serve as the controller of posture and locomotion [6,8,9].

The rat or cat spinal cord isolated from supraspinal control via a complete low- to mid-thoracic spinal cord transection produces locomotor-like patterns in the hindlimbs when facilitated pharmacologically and/or by epidural spinal cord stimulation [10,11]. Thus, locomotor-like patterns can be modulated by stimulation of the networks intrinsic to the spinal cord without the contribution of descending signals. To take advantage of these properties, a more thorough knowledge of the mechanisms of spinal cord stimulation, along with a more detailed understanding about specific sites and parameters of stimulation and their corresponding motor output is needed.

Ichiyama et al. [12] reported that epidural electrical stimulation of the spinal cord can induce rhythmic, alternating hindlimb locomotor activity in chronic spinal rats. Stimulation at the L2 spinal segment at frequencies between 30 and 50 Hz consistently produced successful bilateral stepping. Similar epidural stimulation at other spinal segments were less effective, e.g., epidural stimulation at the T13 or L1 evoked rhythmic activity in only one leg and stimulation at the L3, L4, or L5 produced mainly flexion movements.

More recently, completely paralyzed (motor complete, sensory incomplete) human subjects were implanted with a commercially available spinal cord electrode array and stimulation package originally designed for pain suppression [8], unpublished observations. Stimulation of specific spinal segments (caudal electrodes, ~ S1 spinal level) in combination with the sensory information from the lower limbs and weeks of stand training was sufficient to generate full weight-bearing standing. These subjects also recovered some voluntary control of movements of the toe, ankle, and the entire lower limb, but only when epidural stimulation was present. Thus it appears that the epidural stimulation provided excitation of lumbosacral interneurons and motoneurons that, when combined with the weak excitatory activity of descending axons that were not otherwise detectable, achieved a level of excitation that was sufficient to activate the spinal motor circuits. These results demonstrate that some patients clinically diagnosed as having complete paralysis can use proprioceptive input combined with some synaptic input from descending motor signals, perhaps residual but functionally silent without epidural stimulation to the spinal motor circuits, to generate and control a range of motor functions during epidural stimulation.

These studies suggest that the intrinsic circuits of the spinal cord, if intact, are desirable targets for stimulus-based therapies and strategies. Secondly, the specific stimulation parameters are highly critical to the pattern and quality of functional motor output. The technological hurdles to reach these targets are non-trivial. We have designed an electrode array capable of selectively stimulating specific segments of the rat spinal cord to generate discrete motor responses using a high-density grid of epidural electrodes embedded within a thin-film flexible substrate [13,14]. Although stimulation occurs at the surface level, miniaturization of the electrode contacts and the use of materials specific to our design restrict the effective field of stimulation to a smaller area as compared with conventional wire surface electrodes.

The specificity and high-density features of the electrode array enable us to capitalize on two key features of the spinal cord circuitries that are believed to be essential for rehabilitating posture and locomotion after spinal cord injury (SCI). Firstly, the spinal circuitry can be neuromodulated and the stimulation can be carefully delimited to affect only relevant areas of the spinal cord, thus optimizing the motor outcome. Secondly, as locomotor circuitries are highly plastic and adapt when provided with sensory cues during motor training [2], the density and versatility of the multi-electrode array allows for rapid adjustments of stimulation protocols and adaptations to physiological changes that may occur in the spinal cord over time after injury.

Several design features were taken into account including the flexibility of the array, biocompatibility of the base, and stability of the electrodes for a chronic implant. Parylene C has emerged as an ideal electrode array substrate due to its biocompatibility, insulative properties, flexibility, and tear resistance [15]. The tear resistance of parylene C is large, making the arrays robust to surgical manipulation, as well as to stresses produced in a moving animal [16]. The techniques needed to manufacture these multi-electrode devices are not unprecedented. This is the first time, however, that this technology has been adapted for the express purpose of controlling stimulation at specific sites of the spinal cord in a chronic preparation. Given these basic principles and the results observed in the animal models with conventional wire electrodes [10,17] and from the human subjects with commercially available electrode arrays [8], it seems likely that use of a high-density electrode array could greatly improve the quality of standing and stepping after paralysis.

Rather than attempting to impose exogenous motor commands, this strategy will capitalize on the intrinsic neural control mechanisms of the spinal cord that remain functional post-SCI, enabling the spinal circuits to process sensory input and to serve as the primary source of control. Using this technology, we can selectively and differentially activate distinct neuronal groups distributed throughout the spinal cord, allowing stimulation of specific electrodes on the array to modulate the physiological state of the spinal circuitry so that sensory input can control various hindlimb motor outputs. To examine the potential capabilities of this stimulation system, we used this novel, flexible, high-density stimulating electrode array during the recovery of standing and stepping in adult rats after a complete mid-thoracic spinal cord transection.

## Methods

Data were obtained from adult female Sprague Dawley rats (270–300 g body weight). Pre- and post-surgical animal care procedures have been described in detail previously [18]. The rats were housed individually with food and water provided ad libitum. All survival surgical procedures were conducted under aseptic conditions and with the rats deeply anesthetized (isoflurane gas administered via facemask as needed). All procedures described below are in accordance with the National Institute of Health Guide for the Care and Use of Laboratory Animals and were approved by the Animal Research Committee at UCLA.

Five rats were implanted and tested for the biocompatibility of the implant and stability of the spinal electrodes and stable EMG responses. Once we were satisfied with the stability of the design, a stable array was implanted in one animal to collect chronic physiological data. Due to the complex nature of the fabrication, implantation, and experimentation processes, a limitation of the study is that the standing and stepping data presented in this manuscript are from one animal chronically implanted for 5 weeks. These data will be used as a stepping-stone for future experiments and design modifications.

### Implant fabrication

**Figure 1:**
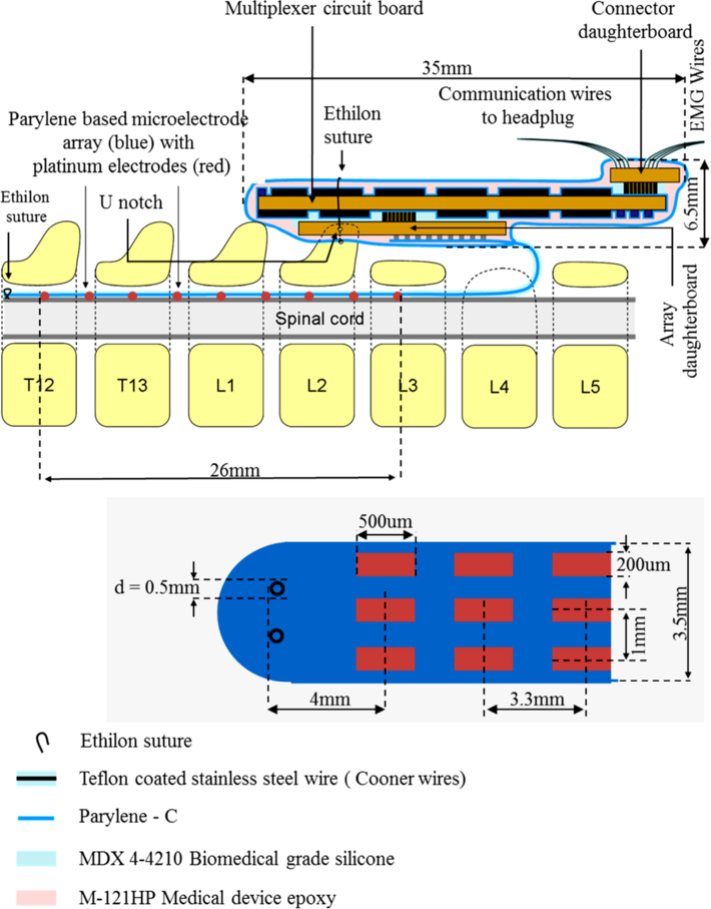
**Experimental design for the parylene-based multi-electrode array.** Parylene based electrode array with multiplexer control and its position and layout with respect to the spinal cord when implanted in the rat. Inset shows the dimensions and design of the platinum electrodes.

**Figure 2:**
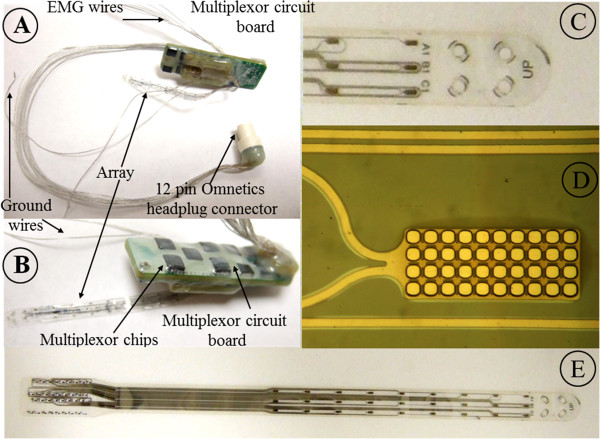
**Dorsal and ventral surface of the multi-electrode array implant and zoomed in view of the electrodes. A**) Ventral view of the implant system: external omnetics connector that is secured to the skull (headplug connector), Teflon coated stainless steel wires from the connector to the circuit board (control wires), electrode array, EMG wires, and ground wires. **B**) Dorsal surface of the implant. **C**) Zoomed in view of the multi-electrode array: note the plantinum electrodes, platinum traces, and the holes used to thread the array during implantation. **D**) Zoomed in view of a single electrode along with the platinum traces. Note the grid-like pattern formed by the parylene on the electrode used to prevent delamination. **E**) Expanded view of the parylene-based array with platinum electrodes.

The electrode array is fabricated with a sandwich structure of parylene-metal-parylene. Parylene-C is a USP class VI biocompatible material and its mechanical properties provide the necessary flexibility to make good epidural contact with the spinal cord. The micro-fabrication process begins with an optional layer of sacrificial photoresist being spun onto a wafer followed by a deposition of 10-μm thick parylene-C. It is patterned to form a structural frame around the outside of the electrode array and is followed by another layer of 5-μm thick parylene-C. The metal layer, patterned using liftoff, was deposited using e-beam evaporation and was composed of a titanium adhesion layer of 100 Å followed by 2000 Å of platinum. The top layer of parylene-C is also 5-μm thick. Openings to expose the metal, formation of the frame, and overall device outline were achieved with oxygen plasma etching. The completed devices were released from the wafer using acetone or water and annealed in a vacuum oven at 200°C for 48 h. The full micro-fabricated device is 59 mm × 3 mm and has a 9 × 3 array of electrodes which are 200 × 500 μm with a parylene grid structure to help prevent delamination (Figures 1 &2).

The complete implant consists of this electrode array, a multiplexer circuit, various wires, and a headplug (Figure 1). The multiplexer circuit routes connections and performs pre-ampification to reduce the total number of headplug wires needed from 37, for a passive implant as seen in prior work by our group [13], to just 12 wires. This design reduces surgery complications and also serves as a stepping-stone for a fully wireless design. The electrode array is interfaced to the multiplexer board with conductive epoxy. The implant then is sealed with 20 μm of parylene, biocompatible silicone (MDX 4–4210), biocompatible epoxy (Loctite M-121HP), and another 20 μm of parylene.

### Control box and multiplexer circuit board description

**Figure 3:**
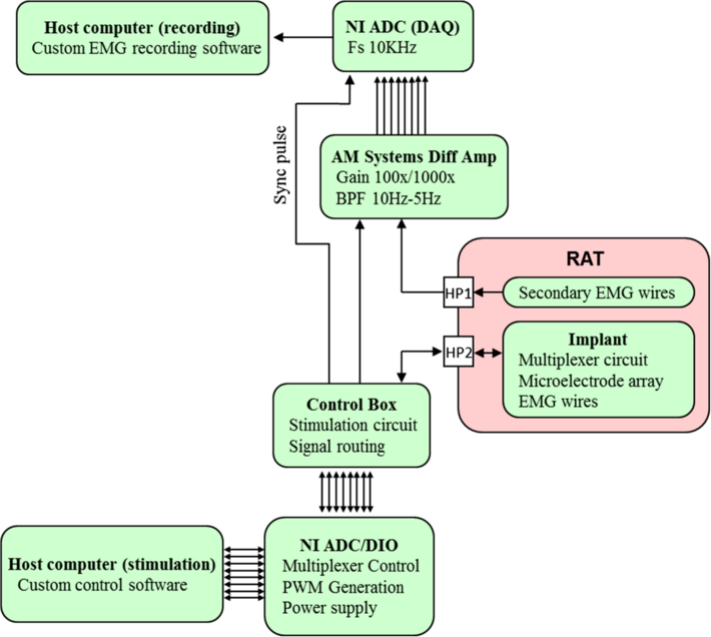
**Block diagram of the experimental setup.** Block diagram showing the experimental setup of the stimulation and recording system. The arrows indicate the direction of the flow of the signals.

**Figure 4:**
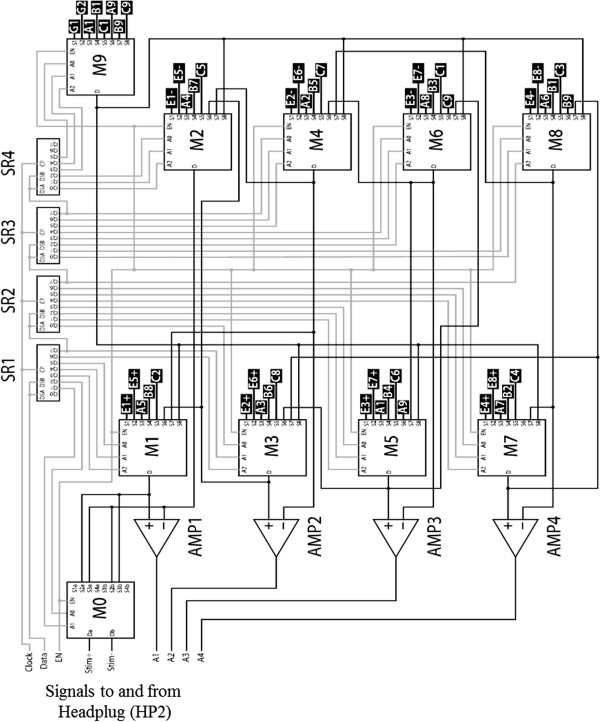
**Schematic of the multiplexer circuit board.** Multiplexer circuit schematic. The 9 lines on the left along with the 3 power lines (12 V, 5 V, and Gnd, not shown) represent the 12 control lines used to interface the array and EMG wires with the external electronics. Black tags represent the spinal cord electrodes and EMG wire pairs.

**Figure 5:**
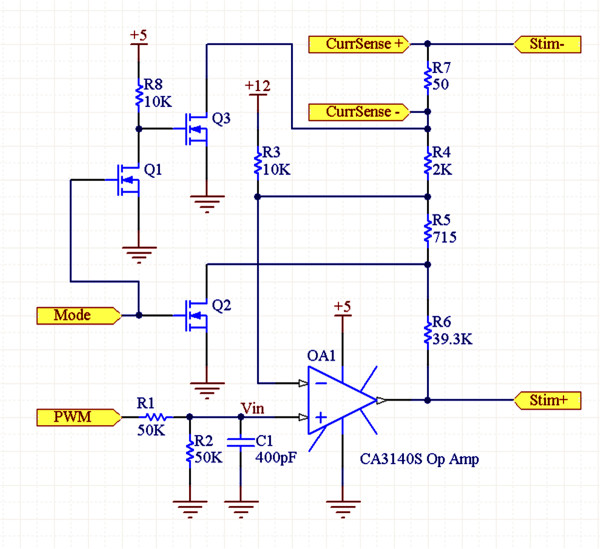
**Schematic of the stimulator circuit board.** Stimulator circuit used describing the use of the Pulse Width Modulation (PWM) to generate the required voltage between *Stim+* and *Stim-*. *Mode* controls current mode vs. voltage mode, and the *CurrSense* signals allow the stimulating host computer to measure the drawn current.

The overall system block diagram is illustrated in Figure 3. The stimulation host computer has a software interface to choose the electrodes to be stimulated along with the stimulation intensity (specified by pulse voltage or current), pulse duration, and pulse frequency. The software generates a 5 MHz signal stream to be output by an ADC/DIO card (National Instruments PXI-6123) and fed to the control box. This signal stream consists of the *EN*, *Clock*, and *Data* signals (Figures 3 &4) to control the multiplexer circuit in the implant, *PWM* (pulse-width modulation) and *Mode* signals for stimulation, and a *Sync* signal to synchronize EMG recordings. The control box has an op-amp circuit (Figure 5) to generate the stimulation signal. The *PWM* signal is passed through an RC filter and creates any required analog waveform at V_in_ (0–2.5 V, ~5 μs effective pulse rise time). When *Mode* is low, the op-amp circuit is transformed to that of a positive gain voltage amplifier (*V*_*Stim+*_ = 25(*V*_*in*_ - 0.86 V)); otherwise, it becomes a voltage controlled current amplifier (*I*_*Stim+*_ = (*V*_*in*_ - 1.92 V)/667Ω). This circuit generates the *Stim+* signal to be fed into the implant’s multiplexer circuit along with the control signals and power lines. The *Stim+* signal also is fed back to the NI ADC for voltage monitoring along with the *CurrSense+* and *CurrSense-* signals for current monitoring. The pre-amplifier signals *A1*-*A4* from the implant pass through a voltage divider (adjustable) and then are output to the EMG amplifier (AM Systems Model 1700). The stimulation signals (*Stim+* and *Stim-)* are fed into the multiplexer circuit that is designed to operate in 4 modes to meet the experimental requirements (both current and future). Current generations: 1) stimulation between almost any two sets of spinal electrodes (bipolar and monopolar) or EMG wires (needed to check position of EMG implants during surgery), and 2) recording from 4 EMG wire pairs. Future generations: 1) recording between multiple pairs of electrodes on the spinal cord, and 2) recording from 4 electrodes in the same column relative to a fifth electrode in the same column (e.g., A1-A9, A3-A9, A5-A9, and A7-A9).

In the multiplexer circuit schematic (Figure 4), the black tag refers to the connection to the spinal electrode. En+, En- refers to an EMG wire pair. A3 refers to a spinal electrode in column A and row 3. G1 and G2 are reference wires (implanted on either side of the back of the animal). Three power lines are present that are used to power up the system: 12 V, 5 V, and ground (not shown in Figure 4). The desired operating mode of the circuit is configured by sending a 30-bit serial data stream (6 μs configuration time) through *Clock* and *Data* that feed into the shift registers SR1–SR4 (NXP Semiconductors 74HC164). These shift registers, in turn, configure the 10 analog multiplexer chips (M0 to M9) and *EN* enables them. M0 (Analog Devices ADG1209) and M1–M9 (Analog Devices ADG1209) are interconnected such that after configuration the desired electrodes or EMG wires are routed either to *Stim+* and *Stim-* during stimulation or to pre-amplifiers AMP1-AMP4 (Analog devices AD8224) during recording. The pre-amplifiers are differential instrumentation amplifiers set to a gain of 200 and send outputs to *A1*–*A4*. The circuit board uses four copper layers and measures 10.3 mm by 33.2 mm.

### Head connector and intramuscular EMG electrode implantation

A small incision was made at the midline of the skull. The muscles and fascia were retracted laterally, small grooves were made in the skull with a scalpel, and the skull was dried thoroughly. Two amphenol head connectors with Teflon-coated stainless steel wires (AS632, Cooner Wire, Chatsworth CA) were securely attached to the skull with screws and dental cement as described previously [12,18]. The medial gastrocnemius (MG), tibialis anterior (TA), and soleus (Sol) muscles were implanted bilaterally with EMG recording electrodes as described by Roy et al. [19]. Skin and fascial incisions were made to expose the belly of each muscle. Two wires extending from the multiplexer circuit board (Figure 1) were routed subcutaneously to each muscle. The wires were inserted into the muscle belly using a 23-gauge needle and a small notch (~0.5–1.0 mm) was removed from the insulation of each wire to expose the conductor and form the electrodes. The wires were secured in the belly of the muscle via a suture on the wire at its entrance into and exit from the muscle belly. The wires were looped at the entrance site to provide stress relief. The proper placement of the electrodes was verified 1) during the surgery by stimulating through the stimulator in the control box (Figures 1, 2, 3 and 4) and by selecting the correct channels on the multiplexer circuit board and 2) post-surgery by dissection.

### Spinal cord transection and array implantation

A partial laminectomy was performed at the T8-T9 vertebral level and a complete spinal cord transection to include the dura was performed at ~T8 spinal level using microscissors. Two surgeons verified the completeness of the transection by lifting the cut ends of the spinal cord and passing a glass probe through the lesion site. Gel foam was inserted into the gap created by the transection as a coagulant and to separate the cut ends of the spinal cord.

**Figure 6:**
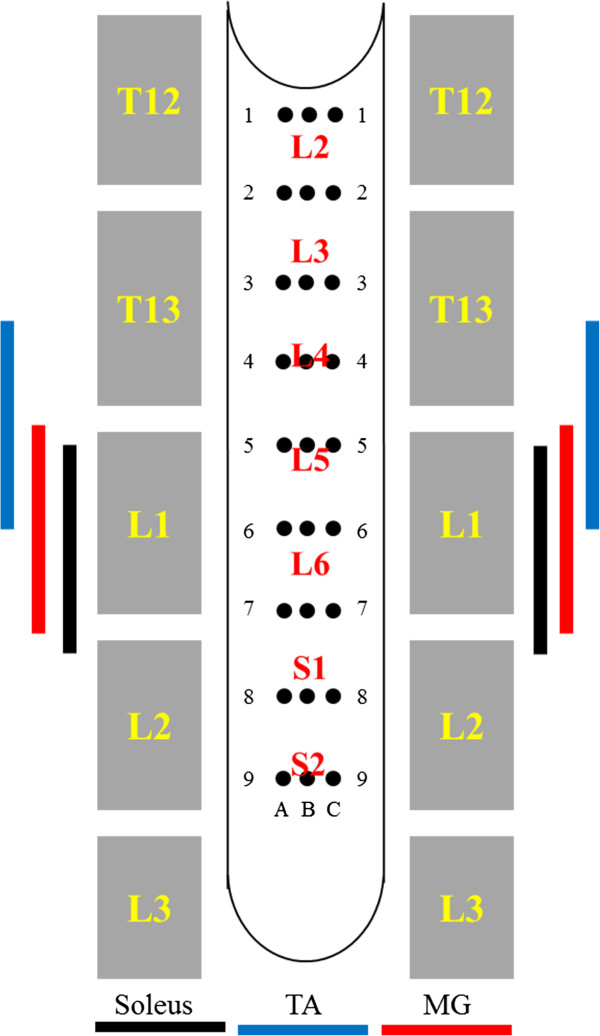
**Location of the motor pools for selected ankle flexor and extensor muscles with respect to the spinal cord level and the sites of electrode implantation.** Vertebral (yellow) and spinal cord (red) levels with respect to the 27 electrodes on the array (black circles) and the location of the motor pools of an ankle flexor (TA, tibialis anterior) and two ankle extensor (MG, medial gastrocnemius, and Soleus) muscles.

To implant the array, the spinous processes and portions of the dorsal and lateral aspects of the vertebrae of T11, and the rostral portions of T12 and L4 were removed. A suture (4.0 Ethilon) was inserted through the opening at T11 and passed down to the opening at L4. This suture then was threaded into holes at the most rostral end of the electrode array (Figure 1 inset) and used to gently pull the array rostrally between the dura and the vertebral column. The most rostral row of electrodes was placed at the middle of the T12 vertebrae. Once the array was positioned satisfactorily over the dorsal surface of the spinal cord, the rostral end of the array was sutured (8.0 Ethilon) to the dura to secure it in position. The L3 spinous process was removed to form a flat surface. The multiplexer circuit board then was placed on the vertebral column over L3. A U notch on the ventral surface of the implant (Figure 1) was secured into the L2 spinous process via a suture (4.0 Ethilon) threaded through a hole on the circuit board and tied around the L2 spinous process. A schematic diagram of the electrode placement and approximate location of the motor pools for the MG, TA, and Sol muscles are shown in Figure 6.

All incision areas were irrigated liberally with warm, sterile saline. All surgical sites were closed in layers, i.e., muscle and connective tissue layers with 5.0 Vicryl (Ethicon, New Brunswick, NJ) and the skin incisions on the back and the limbs with 5.0 Ethilon. All closed incision sites were cleansed thoroughly with warm saline solution. Analgesia was provided by buprenex (0.5–1.0 mg/kg, 3 times/day s.c.). The analgesics were initiated before the completion of the surgery and continued for a minimum of 2 days post-surgery. The rats were allowed to fully recover from anesthesia in an incubator. The spinal rats were housed individually in cages that had ample CareFresh bedding and their bladders were expressed manually 3 times/day for the first 2 weeks after surgery and 2 times per day thereafter. The hindlimbs of the spinal rats were moved passively through a full range of motion once per day to maintain joint mobility.

### Stimulation and testing procedures

Two stimulation protocols were used for testing (Figures 3 &4). A monopolar configuration where the cathode was chosen from one of the 27 electrodes on the array and the anode placed subcutaneously on the side of the body (ground wire, Figures 1, 2, 3 and 4). On the testing day, the cathode was selected sequentially among all electrodes on the array to systematically cover the entire surface of the array and was used to record evoked potentials from the MG, TA, and Sol muscles bilterally. Evoked potentials were recorded from the muscles implanted with EMG electrodes by stimulating the spinal cord at a low frequency (1 Hz) and voltage sweep from 1–8 V (1 V increments) with the rat suspended in a jacket with its hindpaws in contact with a stationary treadmill (bipedal standing position). A bipolar configuration where both the cathode and anode were selected from the set of 27 electrodes on the array was used to facilitate the standing and stepping ability of the spinal rats. Sub–sets of bipolar configurations were tested on different test days. For both the bipolar configurations, the stimulation frequency was based on previously reported values [7,10,12,20,21] and the stimulation intensity was varied (range from 1–8 V) to optimize the standing and stepping ability of the spinal rats. EMG was recorded from the MG, TA, and Sol bilaterally while the rats stepped bipedally on a specially designed motor-driven rodent treadmill at 13.5 cm/s [22]. The treadmill belt had an anti-slip material that minimized slipping while stepping. The rats were placed in a body weight support system that allowed the rat to support the maximum amount of its body weight while stepping with plantar placement.

### Data collection and analysis

EMG recordings from the hindlimb muscles were pre-amplified by the multiplexer circuit board and an external control box before being sent to a band-pass filter (1 Hz to 5 KHz), externally amplified (A-M Systems Model 1700 differential AC amplifier: A-M Systems, Carlsborg, WA), and sampled at a frequency of 10 KHz using a custom data acquisition program written in the LabView development environment (National Instruments, Austin, TX) as described previously [20]. Evoked potentials during standing with low frequency stimulation (1 Hz) were analyzed as described previously [7,10]. The responses were divided into 20 ms windows using the stimulation pulse as the trigger. These windows were averaged over 10 evoked responses and the peak response was detected using custom MATLAB code. These peaks then were binned into early (ER, 1–3 ms latency), middle (MR, 4–6 ms latency), and late (LR, 7–10 ms) responses. The mean amplitudes and latencies for the ER, MR, and LR for both the MG and TA at different intensities of stimulation for each electrode on the array were determined. The EMG signals during weight-bearing standing under epidural stimulation at higher frequencies were analyzed using a custom script written in MATLAB to estimate the MR (latency 4–6 ms) and LR (latency 10–25 ms). The raw EMG signals during bipedal stepping on the treadmill were rectified and then sent through a low pass filter to form a linear envelope to assess the stepping patterns as previously described [23].

### Impedance measurement

A 400 mV sinusoidal wave (10 KHz with a 10 KΩ resistor in series with the spinal electrode and the indifferent ground) was used to test electrode impedance. The voltage across the electrode on the spinal cord and the ground placed subcutaneously in the back region was used to measure the electrode impedance. The electrode impedance was inversely related to the ability of the electrode to stimulate the spinal cord.

## Results

### Facilitation of standing with epidural stimulation

**Figure 7:**
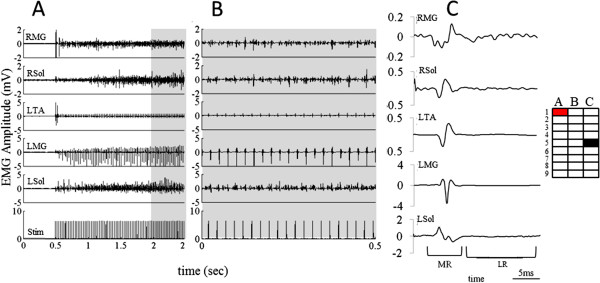
**EMG response to stimulation at rostral electrodes on the array during standing. A**) EMG from ankle flexor and extensor muscles bilaterally while the spinal rat transitions from a crouched to a standing position facilitated by epidural stimulation (40 Hz). **B**) EMG from the right (R) and/or left (L) MG, Sol, and TA muscles during standing under the influence of epidural stimulation (expansion of highlighted region in A). **C**) Average responses of the 20 evoked potentials during full weight-bearing standing under the influence of epidural stimulation shown in B. MR represents the middle response and the LR represents the long latency late response. Note the different amplitude scales for each muscle.

Stimulation of rostral pairs of electrodes at low frequencies (10–15 Hz) produced vibratory movements in both hindlimbs, but did not facilitate standing (Additional file 1: Video 1). Stimulation at higher frequencies (80–100 Hz) resulted in over-activation of the neuronal circuits and produced some non-specific movements in both hindlimbs with no interlimb coordination during standing. In contrast, stimulation between 40–60 Hz resulted in activation of the extensor muscles in both hindlimbs leading to partial weight-bearing standing (Figure 7, Additional file 1: Video 1). Thus, distinct motor responses were induced by stimulation of the rostral electrodes at different frequencies. An example of the motor responses produced by stimulation between electrodes A1 (cathode) and C5 (anode) at 40 Hz is shown in Figure 7 and Additional file 1: Video 1. There is an initial flexion (increased activation of the TA) of the left hindlimb and extension (increased activation of the Sol and MG) of the right hindlimb (Figure 7A). Following this immediate response there is a gradual increase in the level of excitation of the extensors. The intermittent bursting shown in the RMG, RSol, and LSol illustrate the activation of circuitries presumably representing significant levels of polysynaptic activity that are not time-linked to the 40 Hz stimuli (Figure 7B). Additional file 1: Video 1 demonstrates that the right hindlimb initially is bearing greater weight than the left hindlimb. The average evoked responses in selected muscles for 20 stimulations during full weight-bearing standing are shown in Figure 7C. MRs with similar latencies (~5 ms), but varying amplitudes, were observed consistently in all muscles. The RMG shows a higher degree of long latency responses (LR) that may be correlated with the relatively high weight bearing by the right limb.

In contrast to stimulation of rostral electrode pairs, bipolar stimulation of caudal electrode pairs at any frequency failed to facilitate weight-bearing standing. This difference between stimulation of rostral vs. caudal electrode pairs clearly demonstrates the importance of the location of the electrodes and the frequency of stimulation in tuning the neural circuits to generate a specific motor response.

### Facilitation of stepping via epidural stimulation

**Figure 8:**
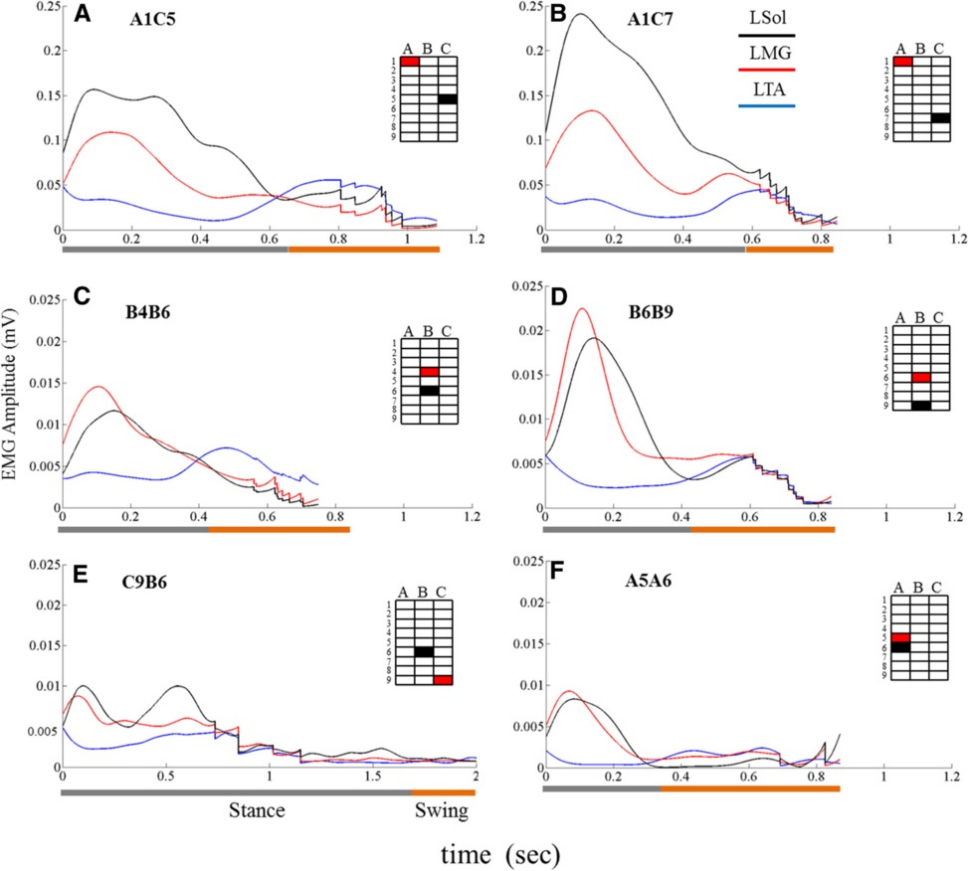
**EMG responses to electrode array stimulation during stepping.** Average (10 consecutive steps) rectified EMG (linear envelope) for an ankle flexor (TA) and two ankle extensor (Sol and MG) muscles during stimulation (at 40 Hz, pulse width 0.2 ms, and 3–4 V) using different electrode combinations. **A** and **B**: coordinated bilateral stepping with good body weight support. **C** and **D**: bilateral stepping with lower body weight support compared to **A** and **B**. **A**, **B**, **C**, and **D**: cases demonstrating good rhythmic bilateral stepping ability with varying degrees of body weight support depending on the position of the cathode and anode on the spinal cord. **E**: Uncoordinated and non-rhythmic stepping during stimulation with the cathode positioned more caudal than the anode demonstrating the importance of having the cathode at a more rostral segment compared to the anode. Note that the time scale for E is the longest due to extended periods of dragging. **F**: rhythmic stepping movements with very low (near zero) body weight support, demonstrating the need to position the cathode and anode at different columns to facilitate stepping with good body weight support. Note the EMG amplitude scale in **A** and **B** are an order of magnitude higher than in **C**-**F**.

The ability of the spinal rats to step with weight support on a treadmill at 13.5 cm/s was tested by stimulating (40 Hz, pulse width of 0.2 ms, and 3–4 V) different pairs of electrodes on the array. The results using 6 different bipolar combinations are shown in Figure 8. Two combinations with the cathode rostral to the anode resulted in coordinated bilateral stepping with good body weight support and interlimb coordination (Figure 8A & B, Additional file 2: Video 2). Two other combinations with the cathode rostral to the anode also produced good bilateral stepping with interlimb coordination, but at a lower body weight support (Figure 8C & D). Thus, stimulation with these 4 combinations of electrodes produced bilateral stepping with good interlimb coordination although the rats had varying weight–bearing capability based on the position of the anode and cathode. In a case where the cathode was placed caudal to the anode and both electrodes were at the caudal portion of the electrode array, the rat was unable to generate weight-bearing stepping (Figure 8E). In another case where the cathode and the anode were placed adjacent on the same column of the electrode array with the cathode placed more rostrally than the anode, the rat was able to generate step-like movements, but with little or no body weight support (Figure 8F and Additional file 3: Video 3).

Combined, these results highlight the importance of the position of the cathode and anode on the spinal cord to facilitate stepping after injury and that the ability to choose between specific sites of stimulation is critical for modulating the types of motor output produced by the epidural stimulation.

### Differential modulation of evoked potentials to low frequency epidural stimulation during standing based on electrode position and stimulation intensity

**Figure 9:**
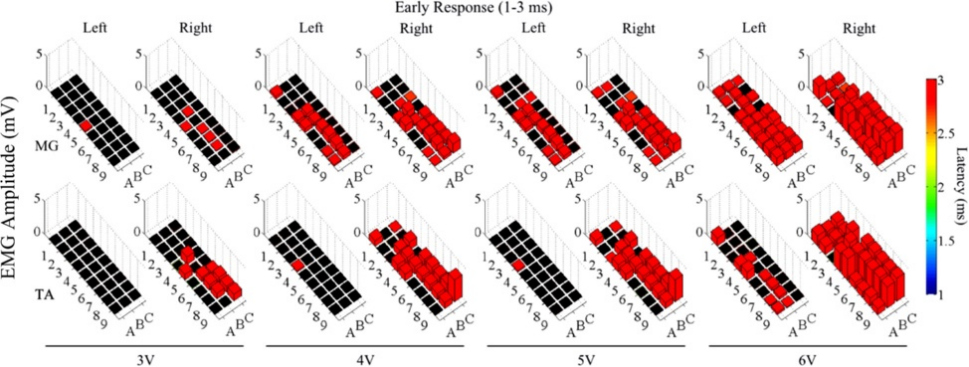
**Effects of low frequency monopolar stimulation on the ER.** Early responses (1–3 ms latency) recorded in the MG (top row) and TA (bottom row) bilaterally during low frequency (1 Hz) monopolar stimulation (3–6 V) at each electrode on the array. The height of each bar indicates the amplitude and the color indicates the latency of the response. The black box indicates a case where no response was recorded for that particular window.

**Figure 10:**
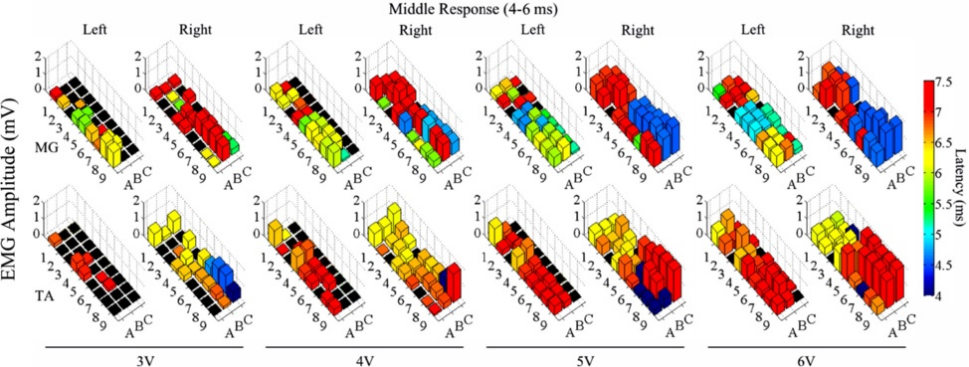
**Effects of low frequency monopolar stimulation on the MR.** Middle responses (4–6 ms latency) recorded in the MG (top row) and TA (bottom row) bilaterally during low frequency (1 Hz) monopolar stimulation (3–6 V) at each electrode on the array. The height of each bar indicates the amplitude and the color indicates the latency of the response. The black box indicates a case where no response was recorded for that particular window.

**Figure 11:**
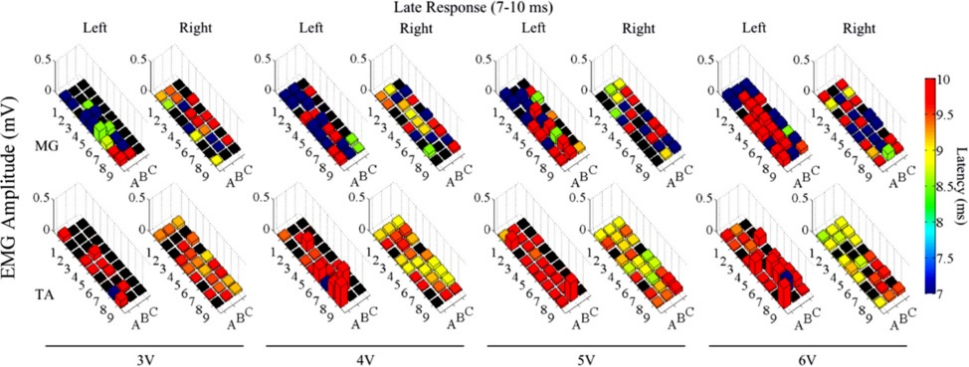
**Effects of low frequency monopolar stimulation on the LR.** Late responses (7–10 ms latency) recorded in the MG (top row) and TA (bottom row) bilaterally during low frequency (1 Hz) monopolar stimulation (3–6 V) at each electrode on the array. The height of each bar indicates the amplitude and the color indicates the latency of the response. The black box indicates a case where no response was recorded for that particular window.

The mean amplitudes and latencies for the ER, MR, and LR for both the MG and TA at different intensities of stimulation for each electrode on the array are shown in Figures 9, 10, and 11, respectively. In general, the ER initially appears around rows 4–6 (Figure 9). Rows 4 and 5 correspond to the beginning of the motor pools for the TA, MG, and Sol muscles (Figure 6), suggesting that the ER may be a direct response to stimulation of afferents without any synaptic delay. As the intensity of stimulation increases, a similar ER (with latency ~3 ms) was observed in rows 1–3 even though these electrodes were not directly over the motor pools of the ankle flexor and extensor muscles. Responses with these short latencies were generally independent of their relative position to the motor pools. The ER amplitudes increased with increased stimulation intensity, consistent with previous results using wire electrodes [7,10]. The increased spatial resolution of the microelectrodes, however, also shows variability across the array, a feature that is not apparent when using wire electrodes.

Similar to the ER, the MR begins around rows 4 and 5 and generally increases in amplitude with increasing stimulation intensity (Figure 10). Unlike the ER, however, the latency of the MR in the TA remains constant from rows 4 to 9 across the stimulation intensities and the latencies in the MG decrease in the more caudal electrodes for any given intensity of stimulation. The MR from stimulation of the most rostral sets of electrodes (rows 1, 2, and 3) shows a much higher latency (~7 ms) compared to the MR from rows 4, 5, and 6 (4–6 ms), i.e., the start of the motor pools of the ankle flexors and extensors, suggesting that there could be an additional synapse for the evoked potential from the rostral region of the spinal cord before the signal reaches the muscles (Figure 10 – e.g., RMG 4 V). The MR in the muscles are higher in the right limb in both the TA and MG at any given voltage (Figure 10 – e.g.,TA and MG at 5 V) through the C, or right side, column of electrodes located ipsilateral to the muscles. Thus, it appears that these evoked responses may be highly dependent on the spatial location of the stimulation. These results highlight the importance of the ability to stimulate specific sites from a therapeutic and device standpoint.

The LR are very general with no real pattern in the observed latencies or amplitudes, suggesting that the LR is a result of activation of various spinal interneuronal circuits that eventually filter down to the muscles. Stimulation at all intensities generates an LR at all electrodes. Several investigators have shown the importance of the presence of an LR to the stepping ability in spinal rats [6-8]. While the specific interaction of the interneurons and the possible structure of these network circuitries are beyond the scope of this paper, it is nonetheless important to identify the diversity of the signals evoked at this level. These results provide important insight into the highly crucial nature of the finite spatial resolution of the stimuli. In addition, the above data indicate that the LR is far less electrode specific than the ER and MR. The functional significance of these observations needs further study.

### Biocompatibility and durability of the chronic multi-electrode array

Electrode impedances were measured daily to assess their reliability and to determine the potential for the array to be implanted chronically. Table 1 shows the average impedance from 5 animals at 1, 3, and 5 weeks post-implantation. Electrode impedances at 7 days post - implantation were similar to impedances recorded *in vitro* (in saline) prior to implantation. At 5 weeks post-implantation only 2-4/27 electrodes were non-functional due to high impedances. Electrodes with higher impedances needed a higher threshold to generate any motor response. Stimulation via these electrodes neither generated any evoked potentials nor facilitated standing or stepping during monopolar/bipolar stimulation. Although electrodes having high impedences (for example in Figures 9, 10 and 11) they did not affect the function of neighboring electrodes i.e. C3,C5 or B4. The spinal cord morphology was assessed (in all five rats) after explanting the array at 5 weeks post-implantation. Neither the array nor the rest of the implant compressed the spinal cord and no signs of infection were observed around the implant. The hindlimb muscles were inspected visually and showed no signs of damage or atrophy beyond that expected after a complete spinal cord transection.

**Table 1:** Average impedances for each electrode in chronically implanted arrays

		**Days Post-Implantation**								
		**7**			**21**			**35**		
		**Array Columns**								
**Array Rows**		**A**	**B**	**C**	**A**	**B**	**C**	**A**	**B**	**C**
	**1**	4.8	5.6	5.9	8.5	11.1	12.8	4.8	13.9	17.0
	**2**	6.6	5.3	8.2	9.2	5.4	9.8	5.2	13.9	17.0
	**3**	8.0	6.7	8.7	5.3	5.0	6.5	3.9	6.8	5.6
	**4**	4.1	9.4	4.0	4.9	6.3	25.9	5.1	18.0	50.0
	**5**	4.1	3.8	6.7	7.7	7.0	7.1	4.4	36.0	7.0
	**6**	5.6	11.5	6.4	4.1	11.6	5.4	4.4	13.0	6.0
	**7**	7.2	4.9	8.9	7.3	6.7	7.4	4.1	8.0	9.8
	**8**	5.8	5.1	4.3	5.2	6.5	6.0	11.2	8.0	7.0
	**9**	5.3	6.1	6.2	7.5	5.8	6.2	9.2	7.0	7.0

Values (k) are the average (n = 5 rats) electrode impedances at 7, 21, and 35 days post-implantation. Increased impedance resulted in higher stimulation intensities needed to evoke a functional motor response. Impedances at 7 days post-implantation were similar to impedances recorded prior to implantation.

## Discussion

We have demonstrated a novel technique, using a high-density parylene-based multi-electrode platinum array, to selectively activate spinal neurons to facilitate standing and stepping in rats after a complete spinal cord transection at a low-thoracic level. The results demonstrate that spinal rats can stand and step when the spinal cord is stimulated tonically at 40 Hz by electrodes located at specific sites on the spinal cord. The quality of stepping and standing was dependent on the location of the electrodes on the spinal cord, the specific stimulation parameters, and the orientation of the cathode and anode. In addition, the amplitude and latency of evoked potentials were determined in non-anesthetized spinal rats during standing to assess the efficacy of selected spinal circuits. The evoked potentials are critical tools to study selective activation of interneuronal circuits via responses of varying latencies.

### Critical features of the stimulation parameters for facilitating standing and stepping

Based on the results, we can generalize that combinations of stimulation with the cathode at the rostral end of the spinal cord results in better stepping ability as compared to combinations with the cathode at the caudal electrodes. This suggests that neurons and neuronal circuits at the rostral end of the spinal cord respond more effectively to the cathode as compared to the caudal sets of electrodes that respond more effectively to the anode. The best results were observed with the cathode and anode located in different rows of the electrode array and the cathode and anode in different columns of the electrode array. The present data also suggest that the more effective standing and stepping can be obtained with bipolar compared to monopolar stimulation. This issue, however, needs to be examined more thoroughly. While the specific composition of the neuronal circuitry and aggregate networks of the spinal cord must be studied further, it is clear that modulating the stimulation protocol and targeting specific anatomical sites of the spinal cord lead to variable motor outputs distinct from one another with unique functional effects.

### Modulation of specific motor pools using the multi-electrode array

The evoked potentials from specific muscles during monopolar stimulation at different intensities allowed us to assess the activation of the motor pools of the ankle flexor and extensors in the spinal cord [24,25]. Evoked potentials from monopolar stimulation reflect the activation of specific neuronal circuits as demonstrated by the responses shown in Figures 9, 10, and 11. Additionally, the higher amplitudes of the MR on the ipsilateral compared to the contralateral side demonstrate the ability to selectively activate different circuitries and to stimulate specific anatomical areas and combinations of motor pools. Different levels of inhibition vs. excitation of spinal circuitries also could be induced selectively. This potential to selectively activate specific combinations of motor pools and levels of inhibition and excitation translates into the unique capability of electrode arrays to control motor behavior.

### Importance of the multiplexer for chronic implantation with wireless capability in small animals

When the durability of an implant is a requirement, the size and biocompatibility of the device are crucial factors in successfully collecting data. Our animal experiments currently rely upon wire bundles to connect the electrode arrays to external computers and electronics. As the number of required connections and the complexity of the device increases, the size of the wire bundle increases as well, reducing the probability of success of the implant due to potential tissue damage and infections caused by the wire bundles. We have partially addressed this problem in our original design by employing a multiplexer (Figure 4) to reduce the number of required connections and changing the form factor of the electrode package into a more easily implantable design. We now plan to develop a relatively generic implantable wireless multi-channel stimulating/recording engine that can be scaled to different species, e.g., rat, cat, or human. This will make the electrode array more useful in a number of ways. For example, the elimination of the wire bundle will increase the biocompatibility of the implant and reduce chances of infection and tissue damage. Additionally, because the wireless system will be a general device designed to have a variety of applications, the transition from animal to human studies will likely be simplified since the fundamental basis of the device will remain consistent.

### Early recovery of stepping and standing after SCI facilitated by epidural stimulation

Several studies have shown that epidural stimulation at L2 and/or S1 using wire electrodes in combination with motor training can facilitate stepping within 3–4 weeks after a complete spinal cord transection [7,9,10,21]. Using the parylene-based platinum electrode arrays described herein we have been successful in facilitating weight-bearing standing and stepping within 8–10 days post-transection. Thus use of the electrode array allows us to tap into the spinal networks to enable stepping sooner after injury as compared to using conventional wire electrodes. Future directions to improve this technology will be to 1) develop computational and mathematical means to detect patterns, determine relationships using evoked potentials, and predict functional outputs, 2) record spinal-evoked potentials during stepping, and 3) combine pharmacological interventions with multi-electrode epidural stimulation as a therapeutic rehabilitation strategy.

### Mathematical modeling to characterize motor responses using learning algorithms

The development of mathematical and computational infrastructures to better characterize motor outputs of stimulation will be crucial to the further development of this neuromodulatory technology. The sheer numbers of involved electrodes, the wide range of stimulation parameters, and the number of functional outcome measures represent a matrix of inputs and outputs that creates a bottleneck to accurately analyze all results. Therefore, it will become necessary to develop tools such as machine-learning algorithms and classification schemes to automate the processing. This is not only important from the perspective of experimental efficiency or basic scientific goals, but particularly from the point of transitioning this technology to clinical therapeutic paradigms. Using a highly differentiated electrode array, it becomes crucial to determine the holistic differences between the smallest variations in the stimulation properties and locations to modulate the networks and produce ordered, desired behavioral outputs. To achieve this, we must develop the means to process and interpret the voluminous information recorded from high-density electrode arrays.

### Need for the ability to record evoked potentials from the spinal cord

The full potential for the use of high-density epidural electrode arrays in clinical and basic scientific studies cannot yet be realized due to limitations in currently available implantable stimulating electronics. The stimulators currently FDA-approved for human studies are too limited in the types of stimulation that they can generate and have no capability to record evoked potentials. Currently, we are unable to detect dynamic changes in intra-spinal cord network interactions during stimulation. The importance of the afferent information to motor command and control cannot be overestimated, yet we have little to no information about the ascending signals that form a significant component of the CPG’s input data. Adding the ability to record from intrinsic networks of the spinal cord could reveal a great deal about the feedback mechanisms that form the foundation for locomotor pattern generation. This will require that the technology for the electrodes be refined to provide optimal characteristics for both stimulation and recording.

### Potential for neuromodulation of the spinal cord and facilitation of specific responses using pharmacological interventions combined with the electrode array

An important aspect of facilitating stepping after SCI is the administration of pharmacological interventions. Although the pharmacological effects are transient, concurrent application of other treatments seems to supplement pharmacologically induced activity [17,20]. These pharmacological treatments appear to raise the excitability of the spinal locomotor circuits by lowering their threshold for activation, and thereby facilitating the effects of multi-electrode epidural stimulation. Specific activation of neuronal networks through the use of an electrode array after administration of pharmacological interventions will allow us to selectively activate specific motor pools for the control of fine movements as well as stepping patterns. Examination of these altered physiological states have the potential to reveal more information about the underlying circuitry of the spinal cord by further delimiting the inhibitory and excitatory components of the circuits responsible for motor behavior, ultimately allowing for the identification and characterization of the neuronal populations responsible for the recruitment of specific motor pools.

### Neurophysiological mechanisms and specific sensorimotor integration impacting motor function via the electrode array after SCI

Given the range of motor behaviors that can be generated with modest levels of stimulation, i.e., primarily sub-motor threshold levels, of different combinations of electrodes and at different frequencies, it is evident that the threshold for excitation of different spinal interneuronal networks are being modulated. Conceptually our strategy for facilitating these motor behaviors is to achieve a physiological state that enables the proprioceptive input derived from stepping and standing to serve as the source of control. That is, the "sub-threshold" intensity of stimulation that modulates the spinal circuitry associated with stepping and standing may not, and actually preferably does not, induce action potentials among the pathways extending from sensory afferents to all of the motor pools. Thus, rather than imposing a specific motor response by stimulating at high intensities, and thus precluding proprioceptive modulation, the activated pathways are determined by the ensemble of sensory information being projected in real time to the spinal circuitry. Regarding the degree of selectivity of specific pathways that could be modulated, it is important to recognize that the extensive divergence of a single Ia fiber from each muscle spindle has extensive synaptic connectivity to not only the homonymous motor pools, but also to synergists and indirectly to antagonistic motor pools through Ia inhibitory interneurons [26]. In addition, robust intersegmental connectivity among the lumbar segments via ascending projections from the sacral segments has recently been reported [27]. Combined, these observations are consistent with the interpretation that epidural stimulation combined with pharmacological modulation is impacting many different pathways simultaneously but in different degrees and proportions based on the stimulation parameters described in the present paper.

## Conclusions

The high density electrode array described in this paper 1) allows high spatial resolution and the ability to selectively activate different neural pathways within the lumbosacral region of the spinal cord to facilitate standing and stepping in adult spinal rats, and 2) provides the capability to evoke motor potentials and thus a means for assessing connectivity between sensory circuits and specific motor pools and muscles. Our initial data underscore the importance of electrode location and anode–cathode orientation and stimulation properties, especially with respect to future therapeutic devices and modulatory “tuning” of epidural stimulation patterns, to provide optimal stimulation for motor function restoration after SCI in animals and humans. Further revisions and additions to this system, including wireless transmission of data, greater software control of the stimulation properties, and increasingly sophisticated data analysis techniques will allow us to further our work/results and gain insights into the neural circuits responsible for specific functional motor responses.

## Competing interests

The authors report no competing interest.

## Authors’ contribution

PG and JC performed the experiments and analyzed the data. MN and YCT fabricated the implant. HZ and RRR performed the surgeries. PG, JC, RRR and VRE wrote the manuscript. All authors read and approved the final manuscript.

## Supplementary Material

Additional file 1: Video 1 This video file demonstrates a spinal rat transitioning from a crouched to a standing posture when facilitated by epidural stimulation (A1C5: Freq – 40 Hz, Amplitude – 3 V, Pulse Width – 0.2ms). Click here for file

Additional file 2: Video 2 This video file demonstrates a spinal rat stepping at 13.5 cm/s on a treadmill with good coordination and body weight support when facilitated by epidural stimulation(A1C7: Freq – 40 Hz, Amplitude – 3.2 V, Pulse Width – 0.2 ms). Click here for file

Additional file 3: Video 3 This video file demonstrates a spinal rat stepping at 13.5 cm/s on a treadmill with minimal body weight support when facilitated by epidural stimulation(A5A6: Freq – 40 Hz, Amplitude – 3.3 V, Pulse Width – 0.2 ms). Click here for file
