# Erratum: Development of a multi-electrode array for spinal cord epidural stimulation to facilitate stepping and standing after a complete spinal cord injury in adult rats

**DOI:** 10.1186/s12984-015-0019-3

**Published:** 2015-03-29

**Authors:** Parag Gad, Jaehoon Choe, Mandheerej Singh Nandra, Hui Zhong, Roland R Roy, Yu-Chong Tai, V Reggie Edgerton

**Affiliations:** Biomedical Engineering IDP, University of California, Los Angeles, CA 90095 USA; Neuroscience IDP, University of California, Los Angeles, CA 90095 USA; Department of Integrative Biology and Physiology, University of California, Terasaki Life Sciences Building, 610 Charles E. Young Drive East, Los Angeles, CA 90095-7239 USA; Department of Neurobiology, University of California, Los Angeles, CA 90095 USA; Department of Neurosurgery, University of California, Los Angeles, CA 90095 USA; Brain Research Institute, University of California, Los Angeles, CA 90095 USA; Department of Electrical Engineering, California Institute of Technology, Pasadena, CA 91125 USA; Department of Mechanical Engineering, California Institute of Technology, Pasadena, CA 91125 USA; Department of Bioengineering, California Institute of Technology, Pasadena, CA 91125 USA

## Erratum

The authors would like to issue an erratum for this article [1], and would like to declare the following competing interests which we inadvertently failed to include in our original publication. The authors would like to apologise for this omission.
